# Internet-based treatment of major depression for patients on a waiting list for inpatient psychotherapy: protocol for a multi-centre randomised controlled trial

**DOI:** 10.1186/1471-244X-13-318

**Published:** 2013-11-26

**Authors:** Jo Annika Reins, David Daniel Ebert, Dirk Lehr, Heleen Riper, Pim Cuijpers, Matthias Berking

**Affiliations:** 1 Division of Online Health Training, Innovation Incubator, Leuphana University, Lueneburg, Germany; 2 Department of Psychology; Clinical Psychology and Psychotherapy, Philipps University Marburg, Marburg, Germany; 3 Department of Clinical Psychology and EMGO Institute for Health and Care Research, VU University Amsterdam, Amsterdam, The Netherlands; 4 GGZ inGeest, Amsterdam, The Netherlands

**Keywords:** Internet therapy, Waiting time, Major depression, Inpatient treatment, Remission

## Abstract

**Background:**

Major depressive disorder (MDD) is a prevalent and severe disorder. Although effective treatments for MDD are available, many patients remain untreated, mainly because of insufficient treatment capacities in the health care system. Resulting waiting periods are often associated with prolonged suffering and impairment as well as a higher risk of chronification. Web-based interventions may help to alleviate these problems. Numerous studies provided evidence for the efficacy of web-based interventions for depression. The aim of this study is to evaluate a new web-based guided self-help intervention (GET.ON-Mood Enhancer-WL) specifically developed for patients waiting to commence inpatient therapy for MDD.

**Methods:**

In a two-armed randomised controlled trial (*n* = 200), the web-based guided intervention GET.ON-Mood Enhancer-WL in addition to treatment as usual (TAU) will be compared with TAU alone. The intervention contains six modules (psycho education, behavioural activation I & II, problem solving I & II, and preparation for subsequent inpatient depression therapy). The participants will be supported by an e-coach, who will provide written feedback after each module. Inclusion criteria include a diagnosis of MDD assessed with a structured clinical interview [SCID] and a waiting period of at least three weeks before start of inpatient treatment. The primary outcome is observer-rated depressive symptom severity (HRSD_24_). Further (explorative) questions include whether remission will be achieved earlier and by more patients during inpatient therapy because of the web-based preparatory intervention.

**Discussion:**

If GET.ON-Mood Enhancer-WL is proven to be effective, patients may start inpatient therapy with reduced depressive symptom severity, ideally leading to higher remission rates, shortened inpatient therapy, reduced costs, and decreased waiting times.

**Trial registration:**

German Clinical Trial Registration (DRKS): DRKS00004708.

## Background

Major depressive disorder (MDD) is one of the most prevalent psychiatric disorders, with a lifetime prevalence that is currently estimated at about 16% [1-3] and predicted to further increase in the foreseeable future [4,5]. By 2030, depressive disorders are anticipated to be responsible for the highest disease burden in high-income countries among all diseases [6]. Suffering from MDD is accompanied by a substantial loss in quality of life, not only for patients but also for their relatives [7-9]. Furthermore, suffering from a depressive episode is associated with a high risk of relapse and recurrence [1,10] as well as a chronic course [11], increased mortality rates [12], significant disability, high medical service use, and major economic costs [13-16].

Although numerous studies provide evidence for the efficacy of available psychological and pharmacological treatments [17,18], many individuals remain untreated [19,20]. The reasons for this treatment gap are twofold. First, individuals who likely to benefit from treatment do not seek treatment because they lack knowledge on available treatment options, anticipate negative (social) consequences, live in underserved areas, fear prohibitive costs, or prefer self-help [20]. Limited availability of clinicians and difficulties in attending therapy during usual business hours are further barriers [21]. Second, those who do seek help rarely receive immediate access to treatment due to long waiting lists for both inpatient and outpatient psychotherapy [20,22]. Consequently, people with an urgent need for therapy are left alone with their burden, which poses several ethical, practical, and therapeutic problems. In addition to high levels of suffering, long waiting times might increase symptom severity and hence increase the need for more intense and longer treatments [23,24].

Another related problem is that because of factors such as limited financial resources and time, inpatient patients are often discharged from the clinic at a very early stage, leaving them with substantial residual symptoms. However, even low levels of residual symptoms are known to increase the risk for relapse and recurrence [25-28]. Recent meta-analyses show that between 40 and 60% of patients with MDD relapse after the initial response to an acute-phase treatment [27].

The use of the internet to provide guided self-help might contribute to solving these problems. First, meta-analytical evidence has shown comparable effects of such interventions compared to traditional psychological treatments when there is at least some support from a professional [29-31]. Furthermore, web-based interventions may represent a far-reaching method for supporting patients during the waiting time for psychotherapy [32].

This method has been well accepted by patients and shown to be effective in the acute treatment phase as well as the maintenance phase [33-37]. The adaptation of internet-based strategies for the waiting phase has several advantages: (a) patients can begin their treatment immediately with no additional waiting time; (b) the online program can be used 24 hours a day, independent of office hours, every day of the week; (c) the material can be reviewed as often as needed; (d) the patient can use it in a familiar environment, without any time or travel costs, which allows for privacy and consistency of care [34,38,39]; and (e) web-based interventions can help patients to practise skills that are relevant for their subsequent therapy (i.e., self-monitoring, problem-solving competencies, behavioural activation).

However, to the best of our knowledge, only one study has evaluated the use of internet-based guided self-help for patients on a waitlist for psychotherapy [40]. In this non-randomised observational study, the researchers found large and significant between-group effect sizes for online treatment for depression (*d* = 0.94) compared to a waiting control group after five weeks. The majority of eligible patients preferred the web-based problem-solving therapy instead of waiting for face-to-face treatment. Participation in this intervention increased the speed of improvement for symptoms of depression during the following therapy [40].

Given the high level of suffering from a depressive episode and the limited availability of psychotherapy resources, new and innovative approaches are needed to address the long waiting times. Using waiting times effectively by providing patients with evidence-based guided self-help methods might result in higher remission rates at the end of inpatient treatment or an earlier remission, thereby reducing costs.

### Trial objectives and purpose

The aim of this multi-centre randomised controlled trial is to evaluate whether a newly developed web-based guided self-help intervention (GET.ON-Mood Enhancer-WL) is effective in reducing depressive symptom severity in patients waiting for inpatient therapy when compared to treatment-as-usual. Moreover, it will be explored whether participants would respond or achieve remission earlier in the course of the subsequent inpatient therapy and would be more likely to be fully remitted at discharge than would controls.

## Methods

### Study design

In this two-armed randomised controlled trial, we will compare GET.ON-Mood Enhancer-WL combined with TAU to TAU alone. The target group will consist of individuals (*n* = 200) who have been referred to inpatient treatment of major depressive disorder by their general practitioner, psychotherapist, or psychiatrist and are awaiting inpatient therapy in one of the German study clinics (Schön Klinik Bad Arolsen/Bad Staffelstein/Bad Bramstedt). The study will be implemented as part of the routine mental health service of the study clinics. Primary and secondary outcome measures will be assessed at baseline, post-treatment (three weeks), at intake for inpatient therapy, weekly during inpatient therapy, and after inpatient therapy. Table 1 gives an overview of the assessments at all planned time points.

**Table 1:** Overview of assessments

**Measure**	**Description**	**Items**	**T**_**0**_	**T**_**1**_	**T**_**2**_	**T**_**3**_	**T**_**4**_	**T**_**5**_	**T**_**6**_	**T**_**7**_	**T**_**8**_	**T**_**9**_	**T**_**10**_
			**baseline pre-treatment**	**weekly monitoring**		**post-treatment**	**pre-inpatient therapy**	**weekly monitoring**					**post-inpatient therapy**
SCID	clinical interview, DSM-V criteria		X										
HRSD primary outcome	depressive symptoms, clinician-rated	24	X			X							
QIDS-C	depressive symptoms and severity, clinician-rated	16	X			X							X
PHQ-9	depressive symptoms	9	X	X	X	X	X	X	X	X	X	X	X
Penn State-worry questionnaire–short version	worrying	3	X			X							X
BA-DS–short version	behavioural activation	9	X			X							X
Social problem solving inventory–short version	problem solving	10	X			X							X
EuroQoL	life quality	5	X			X							X
Brief scale for measuring subjective prognosis of gainful employment	subjective prognosis of gainful employment	3	X										
PATHEV	therapy expectations	11	X			X							X
HAQ	help alliance	11	X			X							X

All procedures were approved by the ethics committee at the Philipps University of Marburg in November 2012 (reference number: 2012-37kr). The trial is registered at the German Clinical Trial Register (DRKS00004708).

### Inclusion and exclusion criteria

Patients will be included in this trial when they (a) are awaiting to be treated with routine mental health care at any of the three study centres, (b) are scheduled to have a waiting time of at least three weeks, (c) are 18 years of age or older, (d) meet criteria of a major depressive episode (according to DSM-V criteria), (e) are motivated to actively participate in the self-guided internet intervention to improve their depressive mood during the waiting time, (f) have access to a computer with an internet connection and a valid email address, and (g) return the signed informed consent form.

We will exclude patients with bipolar disorder or acute psychotic symptomatology (according to DSM-V criteria) or patients showing a notable suicidal risk, as assessed during the structured clinical interview.

### Recruitment

Patients who apply for inpatient therapy via the clinic website (http://www.schoen-kliniken.de/ptp/kkh/bar/anmeldung/krankenhaus/) will be immediately informed about the current trial and can sign up to receive further information. Patients who directly contact the clinic’s head office will receive by mail an information letter that contains the hyperlink to the above-mentioned website.

### Assessment of eligibility and randomisation

**Figure 1:**
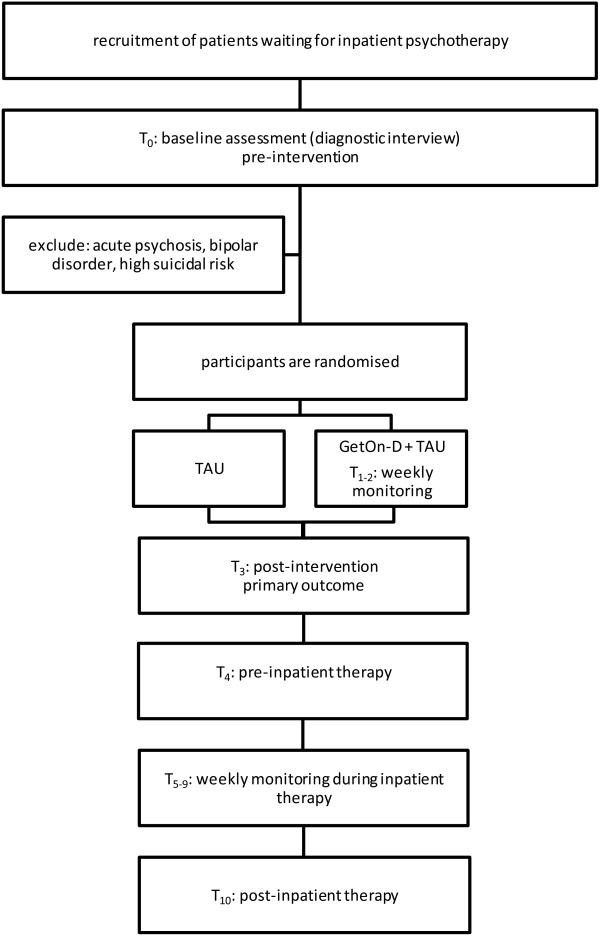
Study flow diagram.

After signing up for further information via the website, prospective participants will receive an email from the research team that includes detailed information about the study. They will be asked to send back an informed consent form via e-mail if they are willing to participate. Patients providing informed consent will be interviewed by telephone to clarify whether they fulfill the inclusion criteria. These structured clinical interviews for DSM disorders (SCID) will be conducted by psychotherapists-in-training who will be weekly supervised by an experienced clinician. Participants providing informed consent, meeting all of the inclusion and none of the exclusion criteria will enter the study. They will be randomly allocated to treatment conditions and complete initial assessment. The allocation will be performed by an independent researcher not otherwise involved in the study using an automated, computer-based random integer generator (randlist). The allocation will be concealed in advance from the participants, researchers involved in recruitment, and inpatient therapists. Patients will be informed that they can withdraw from the study at any time, without any consequences for their subsequent treatment. See Figure 1 for participants’ flow throughout the study.

### Assessments

Self-report and observer-rated assessments will take place at baseline, post-intervention (three weeks after randomisation), at intake for inpatient treatment, weekly during inpatient therapy, and after completion of inpatient therapy (see Figure 1 and Table 1 for a detailed overview). Self-report data will be collected using a secure web-based assessment system (AES, 256-bit encrypted), and observer-based assessments will be conducted via the telephone by trained interviewers. Observer-rated assessments will be recorded to examine interrater reliability. In cases of disagreement, the two raters will discuss until a consensus is formed, and the rating agreed upon will be used for further analyses. If no agreement is reached after discussion, the assessment will be rated by an experienced diagnostic rater, and this rating will be used for analysis.

### Blinding

The research staff conducting the observer-based rating of depressive symptoms will be blinded to the participants’ assigned conditions. Considerable effort is being undertaken to ensure blindness, including the following: (a) an explanation to the participants as to why it is important not to inform the interviewer about the condition to which they were assigned, (b) a written reminder in the interview manual prompting the interviewer to ask the participant not to inform him or her about the randomisation status, (c) verbal reminders to the patient before the interview, and (d) a documentation after the assessment of whether or not the interviewer is still blind to the treatment condition. If the interviewer finds out about the treatment condition another independent interviewer will conduct the interview instead. Participants will not be blinded to the treatment condition.

### Interventions

#### GET.ON-Mood Enhancer-WL–experimental group

The primary purpose of the web-based intervention (GET.ON-Mood Enhancer-WL) is to reduce depressive symptomatology prior to intake for inpatient treatment. In addition GET.ON-Mood Enhancer-WL aims to prepare patients for subsequent inpatient therapy by providing relevant information (psycho education) and using effective and well known elements that are mainly based on behavioural therapy and problem-solving therapy. Abundant evidence exists to support components such as psycho education [18,35], behavioural activation [41,42], and problem solving [43,44] in the treatment of major depression.

A previous meta-regression analysis examining the relationship of treatment intensity to treatment outcome in the treatment of depression indicated that more frequent therapy sessions may be associated with a better outcome compared to less frequent sessions [45]. Thus, we included six modules and advised participants to complete at least one, but preferably two, lessons per week. In all, the training lasts approximately three weeks. We will not include more modules because (a) a recent meta-analysis [46] found web-based interventions for depression including more than seven modules to be less effective (*d* = 0.36) than interventions with seven or fewer modules (*d* = 0 .75) and (b) we wanted to lower the threshold for individuals as much as possible. Table 2 gives an overview of the sessions and their content.

**Table 2:** Overview of content

**Session**	**Content**
Session 1: psychoeducation	First, patients should learn about their disease (symptoms, causes, and types of depression). In addition, the role of motivation in getting through this program should be emphasised.
Sessions 2 and 3: behavioural activation	Second, two behavioural activation sessions will teach the patient about the association between activity and mood. Thus, positive activities can be planned and implemented into the daily routine. In addition, possible obstacles will be considered. The planning of positive activities remains part of the program during the subsequent sessions. Furthermore, participants can work through the facultative module about sleep problems.
Sessions 4 and 5: problem-solving techniques	Sessions 4 and 5 are concerned with problem solving in which the patient learns to distinguish between solvable and unsolvable problems and practises using a six-step plan as a tool to solve problems. Information about unsolvable problems is given as well. Moreover, techniques to stop worrying are introduced.
Session 6: evaluation and preparation	Finally, the patient evaluates his gains and receives a summary of the program. Furthermore, he is encouraged to think about a future plan (such as choosing topics to work on during his inpatient stay).

Given the fact that some studies show a better effect for interventions with at least a low level of guidance compared to unguided interventions [46-48], this strategy might be a promising approach to encourage high treatment adherence. Therefore GET.ON-Mood Enhancer-WL provides a low level of guidance, including individualised feedback for the patient after finishing each of the six sessions. This feedback will be given by psychotherapists-in-training who will receive weekly supervision by an experienced clinician. After completion of the program, there will be no further guidance provided, but the patient will be free to continue with the program or to repeat the exercises.

Some studies have found that highly interactive interventions are more engaging, create higher expectations for improvement, and promote increased motivation and satisfaction in general [49,50]. Hence, the program contains several interactive components for the patients, including a diary to rate their mood and activity level, videos, testimonials, exercises, homework exercises, optional modules, and a library. A strong focus is placed on transfer tasks (homework assignments) to integrate newly acquired strategies and techniques into daily life. In the beginning of each subsequent lesson, participants are invited to reflect on their experiences with the newly acquired skills. The training is adaptive in a sense, as the content is tailored to the specific needs of the individual participant by continuously asking participants to respond by choosing from various response options. Subsequent content is then tailored to the participant’s response. Using responsive web design, participants can follow the program on the internet using a computer, tablet, or mobile phone. An integrated read-aloud function allows participants to follow an audio narration of the lessons.

#### Treatment as usual (TAU)–control group

TAU includes a wide variety of treatment alternatives, such as antidepressant medication, counselling, face-to-face psychotherapy, or no treatment at all. However, to control for potential confounding effects, treatment utilisation as well as changes in the dose of antidepressant medication will be closely monitored during the study period.

#### Inpatient treatment–both groups

Inpatient treatment will generally base on Cognitive Behavioral Therapy (CBT). Patients will receive one to two session of individual therapy (50 minutes) and an average of six sessions of group therapy (90 minutes) per week. Interventions will be supplemented with sports-and physiotherapy, as well as medical treatment (including medication) when necessary. Duration of treatment usually range between 21 and 56 days.

### Sample size

This study will be designed to detect a difference of *d* = 0.40 between the groups as the primary outcome. As patients expect to be treated for their depression in the subsequent inpatient psychotherapeutic treatment, we do expect a somewhat smaller effect size for our intervention than what has been found in the most recent meta-analysis of internet-based guided self-help treatments for depression [35]. With a power of 0.80 in a two-tailed test and *α* = .05, a sample of *n* = 200 participants would need to be included at baseline (calculated using GPower 3.1).

### Primary and secondary outcomes

#### Primary outcome

The primary outcome is depressive symptom severity, as measured by the Hamilton Rating Scale for Depression (HRSD_24_) [51], with assessments conducted pre-and post-treatment. The HRSD is likely the most widely used clinician-rated scale for measuring depression. It assesses depressed mood, vegetative and cognitive symptoms of depression, and anxiety symptoms during the prior seven days. Items are rated on either a five-point or a three-point scale, and the total score is derived by summing the individual item scores. Higher scores indicate greater symptom severity. The HRSD has an interrater-reliability of 0.90 [51] and shows high internal consistency (*α* = 0.88) [52]. Furthermore, it is sensitive to change over time and treatment and corresponds well with overall clinical ratings of severity [53,54]. The cut-off points of 10, 19, 27, and 35 represent the thresholds for mild, moderate, severe, and very severe depression, respectively [52].

#### Secondary outcomes

##### Depressive symptomatology

Observer-based ratings of depressive symptom severity will additionally be collected using the 16-item Quick Inventory of Depressive Symptomatology–Clinician-Rating (QIDS-CR_16_) [52] to achieve a more nuanced understanding of the symptom severity. The QIDS-CR_16_ is a brief clinician-report rating scale developed from the 30-item Inventory of Depressive Symptomatology [55,56]. Unlike the HRSD, the QIDS-CR_16_ evaluates only the nine depression criterion symptom domains (i.e., sad mood, concentration, self-criticism, suicidal ideation, interest/involvement, energy/fatigability, sleep disturbance, appetite/weight change, and psychomotor agitation/retardation) from the Diagnostic and Statistical Manual of Mental Disorders (Fifth Edition) [57] during the prior seven days. Each item is scored on a scale from 0 to 3 points, with higher scores indicating higher symptom severity. This measure has shown good psychometric properties, such as strong internal consistency (*α* = 0.85), concurrent validity, and sensitivity to symptom change in patients with MDD [56]. The cut-off points of 6, 11, 16, and 21 represent the thresholds for mild, moderate, severe, and very severe depression, respectively.

Self-reported depressive symptoms will be assessed with the Patient Health Questionnaire (PHQ-9) [58]. This measure has comparable sensitivity and specificity to many other depression measures, although it is significantly shorter than most other measures [59]. The internal reliability of PHQ-9 reaches values between *α* = 0.86 and 0.89. The score ranges from 0 to 27 because each of the nine items can be scored from 0 (“not at all”) to 3 (“nearly every day”). The cut-off points of 5, 10, 15, and 20 represent the thresholds for mild, moderate, moderately severe, and severe depression, respectively [58,59].

##### Quality of life

Health-related quality of life will be measured with the generic EuroQol [60]. The EuroQol includes the EQ-5D and a visual analogue scale. The EQ-5D consists of five items covering five dimensions (mobility, self-care, usual activities, pain/discomfort, and anxiety/depression). The items are rated as causing “no problems”, “some problems”, or “extreme problems”. This questionnaire shows good psychometric properties in terms of validity and reliability in Germany [61].

##### Problem-solving skills

Problem-solving ability (i.e., generalised appraisal, beliefs, expectancies, and emotional responses) will be measured with two subscales of the Social Problem-Solving Inventory-Revised (SPSI-R) [60]. The positive problem orientation (PPO) subscale represents a constructive dimension, whereas the negative problem orientation (NPO) subscale is viewed as a dysfunctional dimension. Both subscales demonstrate strong psychometric properties. Cronbach’s alphas for these subscales are *α* = 0.76 for the PPO dimension and 0.83 for the NPO dimension [62].

##### Behavioural activation

Participants’ activation towards goals/values and pleasant activities as well as avoidance behaviours will be measured with the BADS-Short Form (BADS-SF) [63]. The BADS-SF includes nine items comprising two subscales (activation and avoidance). The items are rated on a seven-point scale. Higher scores indicate that the individual scores high on the area of interest. The BADS-SF shows good psychometric properties. The internal consistency is *α* = 0.82 [63].

##### Worrying

Worrying will be assessed with the ultra-brief version of the Penn State Worry Questionnaire (PSWQ) [64]. This version consists of three items derived from the standard version, with each item being rated on a seven-point scale. The total score ranges from 0-18, with a higher score indicating more worry. The ultra-brief version shows similar psychometric properties as the standard version and an internal consistency of *α* = 0.85 [64].

##### Therapy expectations and helping alliance

Therapy expectations will be assessed with the Patient Questionnaire on Therapy Expectation and Evaluation (PATHEV) [65], which includes the following three scales: hope for improvement, fear of change, and suitability. The items are rated on a three-point-scale. The reliability of the scales has been shown to be good to sufficient (*α* = 0.73-0.89) [65].

Therapeutic alliance will be measured with the Helping Alliance Questionnaire (HAQ) [64]. This self-report questionnaire contains 11 items rated on a six-point-scale and including two subscales: “helpfulness” and “cooperation”. The scale exhibits excellent internal consistency (*α* = 0.89) and test-retest reliability [66,67].

##### Response remission, and time to remission

Based on the recommendation of Rush (2006), we will use a definition of response/remission that reflects a clinically significant benefit, depending on the specific setting and needs in this trial [68]. A decline in the PHQ-9-score of at least five points will be taken as a clinically significant response. Remission in this specific context will be understood as a symptom-free state, an absolute PHQ-9-score of less than ten will be defined as partial remission, and a PHQ-9-score of less than five will be defined as remission [58]. Time to remission will be defined as the number of days until the symptom-free state is achieved for the first time.

### Statistical analyses

This clinical trial will be conducted in compliance with the protocol of the Declaration of Helsinki and GCP. Aiming at an intention-to-treat design we will include all participants who will be randomly assigned to conditions. Additional per protocol analyses (PPA) will be conducted, including only participants’ satisfying protocol treatment.

Missing data will be handled using multiple imputations (MI). MI is especially robust with respect to missing data [69]. Nevertheless, to assess systematic effects of missing data that cannot be ignored, pattern mixture analyses for multi-level longitudinal approaches [70] will be conducted. To determine whether the treatment effect is dependent on missing data, the missing-data pattern of each participant will first be coded and then included in a three-way interaction (missing-data pattern x condition x change in depression severity) in the main outcome analyses. If no significant interactions between the missing-data pattern and the treatment outcome are found, it can be concluded that it is very unlikely that missing data skewed the results.

The mean improvement scores on all continuous outcome measures will be analysed within and between groups via mixed-model analyses of variance. For all mixed-model analyses, Cohen’s *d*[71] will be calculated by standardising the differences between baseline and follow-up scores by the pooled standard deviation of baseline scores.

The clinically significant improvements between pre-treatment and post-treatment will be examined with regard to the definition of Kroenke (2002) [58]. Differences in response and remission rates will be analysed using chi-square tests. We will also calculate the number needed to be treated (NNT) to achieve response, partial remission, or remission. Time to remission will be measured in days, and the differences between groups in time to remission will be compared using a t-test for independent samples.

Analyses will be performed using an alpha level of 0.05 and one-sided tests will be used for unidirectional and two-sided tests for bidirectional hypotheses. All analyses will be conducted using SPSS 21.

## Discussion

Major depression is a prevalent and severe disorder. Several circumstances lead to the fact that patients remain untreated despite their urgent need for help. Web-based treatments may be an appropriate way of bridging the time gap for patients on a waiting list. Using waiting time efficiently by introducing patients to upcoming therapeutic components before the start of inpatient therapy might be a promising approach to reduce the burden of disease for the patients, to promote earlier discharges and, as a result, to achieve shorter waiting times for subsequent patients.

This study will probably contribute to the literature by evaluating a new web-based intervention for depression compared to treatment as usual in patients on a waiting list for inpatient psychotherapy in a rater-blind randomised controlled trial. We will (a) test the main hypothesis that providing patients access to internet-based, guided self-help will lead to reduced symptom severity before inpatient treatment starts. Moreover, we will (b) investigate whether patients achieve remission earlier and (c) whether we can find significantly more patients with remission or response at the end of inpatient treatment.

There are several strengths to this study: This study will likely be one of the first randomised controlled studies that investigates a web-based intervention for patients on a waiting list for inpatient psychotherapy. We will also (a) use validated clinician-rated measures administered by independent raters blind to treatment condition in addition to self-report, (b) include a specific sample defined by standard diagnostic measures, (c) implement an appropriate statistical analysis plan, and (d) use state-of-the-art methods to cope with missing data.

This trial has also several limitations: As the trial will be conducted within the context of routine mental health care, the time span between the post-treatment assessment (conducted after the web-based treatment) and the beginning of inpatient therapy depends on both the capacities of the clinic and the eagerness of the patient and therefore will vary between patients. We will assess post-treatment data in both conditions at three weeks after randomisation to ensure a comparable time frame. Hence, patients will be at varying states within the online training when the post-treatment assessment takes place. Furthermore, we chose not to include a placebo/attention control condition because the TAU condition cannot be foreknown and is expected to be quite heterogeneous. Besides it is our aim is to study the effectiveness of GET.ON Mood Enhancer-WL in addition to and compared with usual care, as is standard in pragmatic trials [72]. However, due to the lack of such an active control condition, the mechanisms responsible for any possible effects will remain unclear. Last, we will include follow-up assessments only until the end of inpatient treatment. Therefore, we cannot draw conclusions about the effects on the long-term course of symptoms.

In the long term, it would be a desirable step to increasingly implement a new procedure such as this into the daily routine of inpatient as well as outpatient therapy. It would be good practice for the therapist to know in detail what steps the patient has taken previously and to be able to continue the previous gains. This knowledge would assist both the patient and the therapist, saving time and effort, and allowing them to concentrate on further issues and to intensify the treatment process.

Future research could make use of a homogenous control group, focus on various modifications of the program to identify active ingredients, compare different interventions that are likely to be effective as pre-inpatient treatments, evaluate web-based interventions for other disorders than depression or change the setting into the field of outpatient therapy.

## Competing interests

Professor Berking is a minority shareholder of Minddistrict GmbH, which will provide the platform for the web-based interventions.

## Authors’ contributions

MB obtained funding for this study. JR, DE, HR, and MB contributed to the development of the GET.ON depression training. All authors contributed to the study design. JR drafted the manuscript. JR, DE and MB contributed to the further writing of the manuscript. All authors read and approved the final manuscript.

## Pre-publication history

The pre-publication history for this paper can be accessed here:

http://www.biomedcentral.com/1471-244X/13/318/prepub
